# Emergence of Protein Fold Families through Rational Design

**DOI:** 10.1371/journal.pcbi.0020085

**Published:** 2006-07-07

**Authors:** Feng Ding, Nikolay V Dokholyan

**Affiliations:** Department of Biochemistry and Biophysics, University of North Carolina at Chapel Hill, Chapel Hill, North Carolina, United States of America; Harvard University, United States of America

## Abstract

Diverse proteins with similar structures are grouped into families of homologs and analogs, if their sequence similarity is higher or lower, respectively, than 20%–30%. It was suggested that protein homologs and analogs originate from a common ancestor and diverge in their distinct evolutionary time scales, emerging as a consequence of the physical properties of the protein sequence space. Although a number of studies have determined key signatures of protein family organization, the sequence-structure factors that differentiate the two evolution-related protein families remain unknown. Here, we stipulate that subtle structural changes, which appear due to accumulating mutations in the homologous families, lead to distinct packing of the protein core and, thus, novel compositions of core residues. The latter process leads to the formation of distinct families of homologs. We propose that such differentiation results in the formation of analogous families. To test our postulate, we developed a molecular modeling and design toolkit, Medusa, to computationally design protein sequences that correspond to the same fold family. We find that analogous proteins emerge when a backbone structure deviates only 1–2 Å root-mean-square deviation from the original structure. For close homologs, core residues are highly conserved. However, when the overall sequence similarity drops to ~25%–30%, the composition of core residues starts to diverge, thereby forming novel families of protein homologs. This direct observation of the formation of protein homologs within a specific fold family supports our hypothesis. The conservation of amino acids in designed sequences recapitulates that of the naturally occurring sequences, thereby validating our computational design methodology.

## Synopsis

Studies of known proteins have revealed intriguing co-organization of their sequences and structures. Proteins with sequence similarity higher than 25%–30% usually adopt a similar structure and are called homologs, whereas those with low sequence similarity (<20%) can share the same structure and are referred as analogs. The origin of such co-organization has been a topic of extensive discussions among protein folding, design, and evolution research communities, because understanding of the emergence of homologs and analogs in the protein universe has broad implications for our ability to rationally manipulate proteins. In this study, the authors developed a molecular modeling and design method, Medusa, to computationally design diversified protein sequences that correspond to similar backbone structures, which determine a protein fold family. Using Medusa, the authors directly demonstrated the formation of distinct protein homologs within a specific fold family when the structure deviates only 1–2 Å away from the original structure. The study suggests that subtle structural changes, which appear due to accumulating mutations in the families of homologs, lead to a distinct packing of the protein core and, thus, novel compositions of core residues. The latter process leads to the formation of distinct families of homologs.

## Introduction

Understanding the evolution of proteins is an intriguing but challenging problem in molecular biology [1–12], which in many regards is vital to the progress in the field. One of the puzzling observations about the zoo of known protein structures, which emerge as the direct result of evolution, is the limited number of occurring species, even by very conservative estimates [13]. What is more surprising is that multiple distinct protein sequences can share the same three-dimensional structure. Generally, proteins that share at least 25% sequence similarity form families of homologs (also known as fold families) [14–18]. Proteins from distinct fold families, with little sequence similarity, but similar structures, constitute families of analogs (also known as superfold families) [12,19,20]. While it is now clear that the creation of new folds may not be as difficult as it was initially believed [21], it is unclear why a small fraction of possible protein fold space is explored in nature, limiting the zoo to only few species.

A plausible explanation to a limited usage of possible protein structures is that the diversification of this zoo is not under selective pressure. Instead, the reusability and adaptation of protein structures to emerging functions is a feasible mechanism for adapting to an ever-changing environment. Hence, protein functional plasticity shapes the zoo of protein structures. Indeed, taking into consideration the thermodynamic stability of protein folds in a statistical evolution model, Dokholyan and Shakhnovich [22] demonstrated that protein analogs and homologs can originate from the same ancestor. The authors demonstrated that distinct protein fold families within a superfold family diverge in their evolutionary time scales, as a consequence of the physical properties of the protein sequence space [22]. However, the simplicity of the protein model employed in reference [22] did not permit direct observation of the emergence of protein homologs and analogs and detection of the sequence-structure relationships that dictate the differences between these families.

Here, we postulate that subtle structural changes, which appear due to accumulating mutations in the homologous families in a course of evolution, lead to distinct packing of the protein core and, thus, novel compositions of protein core residues. The latter process leads to differentiation into distinct families of homologs and, ultimately, the formation of families of analogs. To test this postulate we developed a molecular modeling and design suite, Medusa, which permits simultaneous exploration of protein sequence and structural space. A detailed all-atom representation of proteins and a parameterized force field in Medusa allow us to accurately score amino acid substitutions and explore structural perturbations associated with mutations.

In principle, we can use Medusa to explore the evolution of a protein fold family in a dynamic manner, i.e., by monitoring the time-dependent sequence and structural changes upon random mutations of amino acids [22]. However, because such a study is extremely time-consuming using a high-resolution protein model [23], previous investigations were limited to generating thermodynamically stable sequences through successive mutations in a protein for a given structure [24–28]. Due to strong stearic restrictions in the core of globular proteins, sidechain packing in a redesigned protein is often very similar to that of the native protein, i.e., the redesigned proteins are close homologs of the native protein [24,27–29]. Hence, it is important to introduce perturbations within the conformational space, for modeling the diversity of sequence space in the protein superfold family.

The introduction of small, random perturbations in backbone dihedral angles has been proposed as a method to model the sequence diversity in protein homologous families [30,31]. Because such a random sampling approach does not have any energetic guidance, this method is limited in its ability to reproduce the sequence diversity of protein fold families [32]. To circumvent this limitation, Baker and coworkers [21,32] proposed to use the same energy function to guide the structural search as the one used in sequence optimization for a given structure. In these studies [21,30–32], the structural search is usually achieved by perturbations of a randomly chosen backbone dihedral angle, followed by complimentary adjustments of a set of successive dihedral angles to avoid large deviations of the rest of the structure. However, this type of structural search algorithm has intrinsic limitations, because the Monte Carlo move set may impose structural biases during the search. Here, we propose to use a fast dynamic algorithm, discrete molecular dynamics (DMD [33–37]), to sample protein conformational space. Conformations obtained from DMD automatically satisfy fold constraints, while allowing both local and global perturbations.

Combining Medusa and DMD in an evolutionary model of protein fold families (Medusa/DMD), we generated a diverse ensemble in the structural vicinity of reference proteins (using DMD) and found corresponding thermodynamically stable sequence for each generated structure (using Medusa). We found that analogous proteins emerged when the backbone structures depart only 1–2 Å root-mean-square-deviation (RMSD) from the reference structure and that core residues in close homologs were highly conserved. We also observed that only around a critical sequence similarity range of ~20%–30% (the empirically observed “twilight zone” that differentiates protein homologs and analogs [38,39]) did the protein core diverge as much as the rest of amino acids. This observation supported our hypothesis that the amino acid composition of a protein core determines homology. Importantly, our simulations reproduced the empirically observed range of the “twilight zone.” In addition, amino acid substitution/conservation profiles of homologous proteins from our model recapitulated that of the naturally occurring homologous proteins taken from the database of homology-derived secondary structure of proteins, HSSP [14]. Hence, Medusa/DMD methodology is a viable approach for exploring protein sequence-structural space that can be utilized for evolutionary studies.

## Results

We identified the low-energy sequence and structure for a given fold by iteratively performing sequence optimization for a fixed backbone (also known as the “inverse folding problem” [40,41]) and structural optimization for a given protein sequence [21,32]. The native sequence recapitulation rate is an indicator of the performance of a protein design approach [21,32,42]. Hence, we first tested the ability of our sequence design method, Medusa, to recapitulate native protein sequences given their backbone structures.

### Recapitulating the Native Sequences with a Fixed Backbone

A protein design method has two primary components: an energy function to evaluate the fitness of a particular sequence for a given structure, and a search procedure to scan through the sequence space [43]. Medusa uses similar energy terms (Materials and Methods) as RosettaDesign, a method developed by Kuhlman and Baker [28] and validated experimentally [21]. Briefly, we used a Monte Carlo-based simulated annealing procedure to identify the optimal sequence for a given backbone structure. To search the sequence space rapidly, rotamer libraries were used to model the amino acid sidechains [44]. A rotamer library contains a discrete set of conformations for each amino acid and is developed to best represent common conformations observed in the protein databank (PDB). For each rotamer, there are associated dihedral angle variations with the standard deviations tabulated in the rotamer library [44]. A small deviation of the sidechain from the average dihedral angles might result in a different energy [28,45]. The major differences between Medusa and RosettaDesign include the sampling algorithm used to search the sub-rotameric space and, in turn, the weights parameterized for different energy terms (Materials and Methods, Protocol S1, and Table S1).

Expansion of the rotamer library by additional division in the sub-rotamer space has been proposed to successfully model the strain of a rotamer conformation [32], i.e., possible clashes between the sidechain and the fixed backbone. Such an expansion of the rotamer library often results in a large dataset, which in turn decreases the efficiency in searching for the optimal sequence. Alternatively, a flexible sidechain redesign protocol (Materials and Methods), in which each trial substitution is followed by a minimization step in the sub-rotameric space, can also release the strain of the trial rotamer with respect to the fixed backbone. Here, we proposed a new sidechain sampling algorithm, stochastic sidechain redesign (Materials and Methods), to model the sidechain flexibility. Next, we compared the performance of these two fixed-backbone design methods in recapitulating native amino acid sequences.

#### Stochastic sidechain redesign out-performs flexible sidechain redesign.

**Figure 1:**
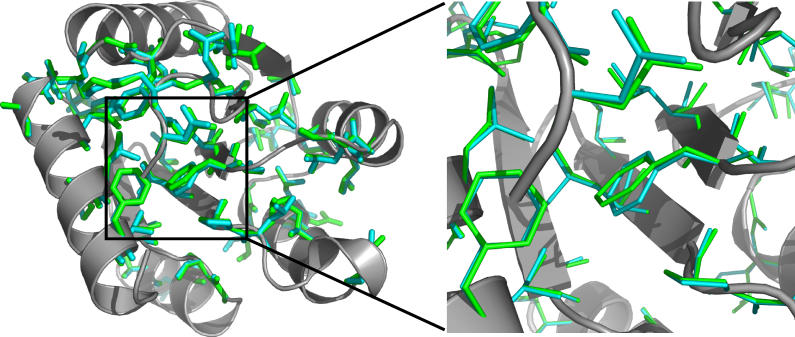
The Native (Green) and Redesigned (Cyan) Chey Protein (PDB: 3chy), Using Medusa Fixed-Backbone Redesign The backbone structure is shown in cartoon, and the sidechains of recapitulated residues are shown in stick representation.

We tested the average sequence recapitulation rate for a set of high-resolution protein structures using the two sidechain sampling methods above: stochastic and flexible sidechain redesign. The test dataset consists of 38 high-resolution protein structures determined by x-ray crystallography. Using different protocols in Medusa fixed-backbone simulations, we obtained distinct native sequence recapitulation rates: 1) 37.9% (63.4%) for stochastic sidechain redesign; 2) 35.4% (55.1%) for flexible sidechain redesign. Here, the sequence recapitulation rates for the whole sequences and for protein cores (Materials and Methods) are shown, and the latter are in brackets. Therefore, the stochastic sidechain redesign out-performs the flexible sidechain redesign in term of recapitulating native sequences. These results suggest that our stochastic sidechain redesign protocol efficiently samples the sub-rotameric space. The better performance of the stochastic sidechain redesign against the flexible sidechain redesign is probably due to the fact that local minimization of a trial rotamer does not guarantee the global minimum: in a flexible sidechain redesign, a non-native amino acid rotamer can be easily trapped during the minimization process. Most importantly, the performance of the stochastic sidechain redesign method, in terms of native sequence recapitulation, is comparable to that of the RosettaDesign study with an expanded rotamer library [32]. In Figure 1, we present the native and redesigned chey protein (PDB code: 3chy) using the stochastic sidechain redesign. The core residues are recapitulated with a high probability and the sidechain rotamers are also reproduced in most cases.

In terms of the computational efficiency, the stochastic sidechain redesign method is also superior compared to flexible sidechain redesign. In the flexible sidechain redesign method, the minimization of each trial sidechain rotamer is computationally expensive. For the same protein, stochastic sidechain redesign consumes approximately one-tenth of the CPU time used by flexible sidechain redesign. Therefore, we employed in Medusa the stochastic sidechain searching algorithm.

#### High native sequence recapitulation rate is not the result of amino acid preferences.

To test whether the high native sequence recapitulation rate is simply due to the backbone-dependent amino acid preference terms in Equation (1) (the internal energies *E_φ,ψ|aa_* and *E_φ,ψ,aa|rot_*, Materials and Methods and Protocol S1), we perform control simulations by turning off various terms in energy calculation. To turn off an energy term, we set the corresponding weight, *W* = 0. We find that sequence preferences and the non-specific van der Waals (VDW) terms alone (*W_solv_* = 0*,W_HB_* = 0*,W_ref_* = 0) dramatically reduce the native sequence recapitulation rate (Table S2), suggesting that the high sequence recapitulation rate is not simply the result of amino acid preferences. A similar decrease of the native sequence recapitulation rate is also observed if we turn off the solvation (*W_solv_* = 0), solvation and hydrogen bond (*W_solv_* = 0,*W_HB_* = 0), and solvation and reference (the unfolded state) (*W_solv_* = 0,*W_ref_* = 0) terms, respectively. Importantly, excluding the sequence preference terms (*E_φ,ψ|aa_* = 0, *E_φ,ψ,aa|rot_* = 0) also leads to a weak native sequence recapitulation rate. Therefore, all energy terms in Equation (1) are equally important to describe interactions in a protein, and thus contribute to the high performance in recapitulating the native sequences. Using the same parameterized force field, we also find a significant correlation between the Medusa-predicted stability changes upon mutations and their experimental values for several proteins (FD and NVD, unpublished data).

### Modeling the Backbone Flexibility with the Backbone Relaxation

We performed DMD simulations to generate homologous backbone conformations as described in the Materials and Methods section. We first performed fixed-backbone redesign simulations for the DMD-generated backbone structures. However, we found that the redesigned sequences have higher energies and lower sequence identities to the native sequence (non-designability), compared to redesigned sequences using an x-ray structure (unpublished data). Similar observation has been reported by Kuhlman and Baker [28] for sequences redesigned from NMR structures. The authors postulated that possible strains in the backbone conformation from the above sources (i.e., NMR or DMD simulations) might lead to the non-designability in a fixed-backbone redesign simulation. To release the strain in the backbone structure, we iteratively perform fixed-backbone sequence redesign and structure relaxation to engineer an optimal sequence and structure for a starting conformation (Materials and Methods). Next, we test the flexible-backbone redesign method on a histidine-containing phosphocarrier protein, the HPR domain (PDB: 1poh [46]).

Using DMD simulations, we generate an ensemble of homologous backbone conformations (~300 conformations) within 2.5 Å RMSD from the initial crystal structure. For each conformation, we performed Medusa redesign simulations with backbone relaxation. We found that Medusa simulations can quickly release the strains in the structure and, thus, reduce the system's energy (a typical redesign simulation is shown in Figure S1). Typically, after approximately 20–50 iterations of sequence redesign and structure relaxation, the energy of the redesigned sequence-structure rapidly reached a plateau, comparable to the redesigned energies using x-ray structures of proteins with a similar length. Similarly, we found that the RMSD between the relaxed conformation and the initial structure converges in a similar manner. The RMSD between the initial and the final structures is usually smaller than 1 Å. In some rare cases, the RMSD can be as large as 1 Å, which suggests that this simple iterative algorithm is efficient in relaxing the strain in the initial backbone conformation.

#### Recapitulating the native sequence entropy using flexible-backbone redesign.

To model the evolution of protein fold families, it is important to know whether the generated homologous sequences from simulations can reproduce the extent of the amino acid substitution/conservation at each position in the corresponding homologous family. The sequence entropy (Materials and Methods) is often used to quantify the degree of amino acid conservation in a homologous family. The lower the sequence entropy, the higher the conservation of the residue's identity throughout evolution. We computed the native sequence entropy of a homologous family from the HSSP database [14]. To calculate the in silico sequence entropy, we first found, for each DMD-generated conformation, the optimum sequence using the flexible-backbone redesign method. To limit the calculation on homologs, we only selected the redesigned sequences with a sequence identity higher than 30%. For consistency, one would like to compute the sequence identity of a redesigned sequence with respect to a reference sequence that is optimized using the same force field. Therefore, we used the optimal sequence, obtained from a fixed-backbone redesign using the x-ray crystal structure, as the reference sequence. We studied three different proteins: HPR domain, ROSSMAN fold (PDB: 3chy; [47]), and SH3 domain (PDB: 1cka; [47,48]).

#### HPR domain.

**Figure 2:**
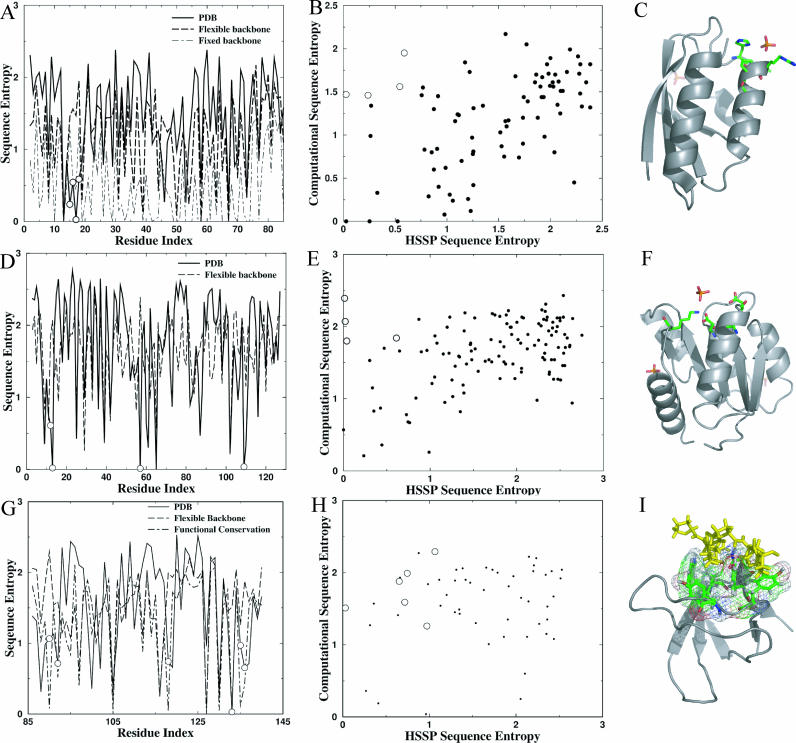
The Sequence Entropy Computed from Simulations versus the Naturally Occurring Sequence Entropy Computed from HSSP Three families of protein homologs were studied: HPR domain (A,D,G), ROSSMAN fold (B,E,H), and SH3 domain (C,F,I). The open circles (○) in (A–F) correspond to the functionally important residues. In (G–I), these functionally important residues are shown in stick representation. In (G,H), the SO_4_^2−^ ions are used to mimic the phosphate anion in crystal preparation. In (I), the poly-proline peptide are shown in yellow and the peptide-binding residues form a continuous surface, shown in mesh representation.

We present in Figure 2A the native and in silico sequence entropy as a function of residue index for the HPR domain. Interestingly, we found that the simulated sequence entropy follows a similar trend to the native sequence entropy. There are several large deviations: residues 15–18 and residues around 45 have low native sequence entropies but high sequence entropies from simulations. We found that the overall correlation is around 0.46 including all the residues (Figure 2B). Interestingly, we find that residues 15–18 (marked by ○ in Figure 2A and 2B) correspond to a highly conserved phosphate-binding site in the homologous family [46]. In Figure 2C, we show these residues, which bind the SO_4_^2−^ anion in the crystal structure (during the crystallization, SO_4_^2−^ is used to mimic the phosphate anion). Since our model only takes into account the thermostability, it is expected that we can not capture the amino acid conservation [47,49] due to either function or kinetics. Strikingly, after we exclude these functional residues 15–18, we find the correlation coefficient between HSSP and computational sequence entropies increases to 0.62 (*p* ≈ 1.0 × 10^−9^). For control, we also generated 300 sequences using the fixed-backbone redesign method and compute the corresponding sequence entropy. The sequence entropy computed in this way is much smaller than the native value (Figure 2A), which is simply due to small amino acid variations with a fixed backbone.

#### ROSSMAN fold.

We used the chey protein from Escherichia coli as the reference structure [46] for the ROSSMAN fold. Similar to the HPR domain, we found that, overall, the computational sequence entropy agrees with the HSSP sequence entropy, except for several large deviations (Figure 2D and 2E). Functionally, the chey protein has a conserved cavity on the surface for binding ATP and the subsequent phosphorylation of residue D56. Residues 11, 12, 56, and 108 (Figure 2F) are responsible for the ATP binding. Therefore, these residues have low HSSP sequence entropies in the homologous family, whereas simulations predict high values due to their exposure to the surface (Figure 2D and 2E). After we excluded these functionally-conserved residues, we found the correlation coefficient between the simulated and native sequence entropies increased from 0.40 to 0.53 (*p* ≈ 7.2 × 10^−10^).

#### SH3 domain.

The SH3 domain is a molecular-recognition module that functions by interacting with proteins containing a poly-proline (P) sequence motif (PPXP). The binding to poly-proline requires a set of unusually conserved residues on the surface: residues 6, 8, 34, and 49–51 (Figure 2G–2I). Most of these peptide-binding residues are aromatic. In addition, residue L24 has been shown to be important for the folding kinetics of the SH3 domain and is also highly conserved [49,50]. However, even after we excluded these residues, the correlation coefficient increases from 0.15 to only 0.23 (Figure 2G and 2H). We postulate that the low correlation is due to the fact that poly-proline peptide binding requires a large set of conserved aromatic residues to function, and the packing between the conserved residues and their neighboring residues imposes a strong evolutionary pressure to preserve these neighboring residues. This secondary evolutionary pressure might lead to a high conservation for these residues and, thus, low sequence entropy (i.e., the N-terminal residues in Figure 2G). Therefore, we propose to “conserve” the function during the Medusa flexible-backbone redesign by limiting the available amino acids at the poly-proline-binding positions to the native amino acids, mainly aromatic residues. Interestingly, we found that the correlation coefficient between the new data and the native data increases to 0.53 (*p* ≈ 3.1 × 10^−5^) (Figure 2G).

### Direct Observation of the Emergence of Analogous Proteins

**Figure 3:**
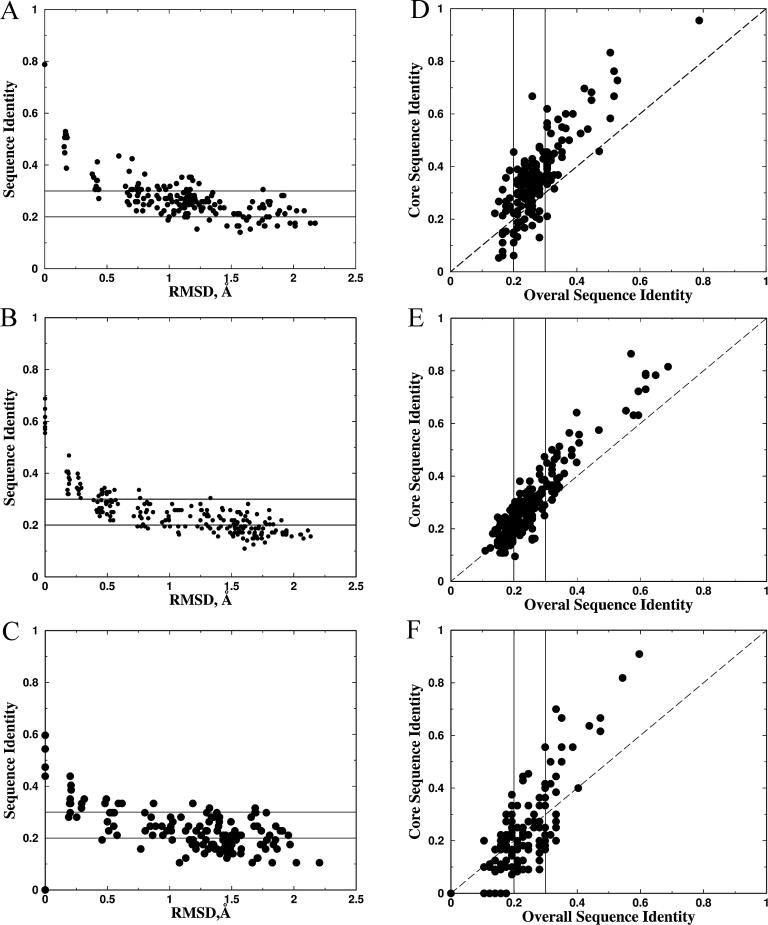
The Sequence Identity for the Constructed Homologous Structures Three different protein folds are studied: HPR domain (A,B), ROSSMAN fold (D,E), and SH3 domain (G,H). (A,C,E) The sequence identities of the redesigned proteins using the flexible-backbone design simulation are presented as the function of the backbone-RMSD from the reference protein. (B,D,F) The sequence identity of the core is also plotted against the overall sequence identity. The “twilight zone” of sequence identity (20%–30%) corresponds to regions between horizontal (A,C,E) or vertical (B,D,F) lines.

Using the flexible-backbone redesign method, we constructed for each reference protein, a new ensemble of stable sequences sharing similar backbone structures to the reference protein. Next, we studied the change in sequence identity as a function of structural deviation with respect to the reference structure. As discussed above, we used the optimal amino acid sequence—obtained from the fixed-backbone redesign on the reference x-ray structure—as the reference protein. In Figure 3A–3C, we present the sequence identity as a function of the RMSD with respect to the corresponding reference proteins for the three different folds: HPR domain, ROSSMAN fold, and SH3 domain, respectively. In general, we found that overall sequence identity decreases as the backbone RMSD increases. The backbone RMSD does not need to be large in order for the emergence of analogous sequences, empirically defined by sequence similarity less than 20%. We find that analogous proteins emerge when the backbone structures deviate only approximately 1 Å RMSD from the reference structure. Then, the question is “what leads to the emergence of analog proteins?” We postulate that accumulative mutagenesis of core residues perturbs the core structure and leads to the development of non-homologous sequences. Next, we studied the relationship between the sequence conservations in the core and overall.

In Figure 3D–3F, we present the sequence identity of the core residues versus the overall sequence identity for the constructed sequence-structure ensembles. The core sequence identity is always higher than the overall sequence identity for homologous proteins, which is consistent with the general experimental observation that packing of core residues is the main stabilizing interaction in protein structures, and substitution of core residues often destabilizes the protein. However, when the protein sequence similarity enters the “twilight zone” (20%–30%), the core residues diverge as much as the rest of the protein in terms of the sequence similarity. For protein analogs (sequence identity <20%), we found that the sequence similarity of core residues was often lower than that of the overall sequence, suggesting radical rearrangement of protein core residues. Therefore, sequence identity of protein core residues is the dominating factor that dictates protein homology, an observation that was made in previous lattice studies [51,52].

## Discussion

Inspired by recent success in computational protein design [21,24–26,29], we developed a protein evolution model combining large-scale structural sampling (using DMD) and protein sequence redesign with backbone relaxation (using Medusa). By testing our Medusa/DMD method on several proteins with different folds, we found statistically significant correlation between the computational and natively-occurring sequence entropies. Our method ranks the redesigned sequences according to their thermodynamic stabilities, which correspond to the major evolutionary pressure [22]. However, it is still a challenge to recapitulate the native sequence substitution/conservation profile, since it is not clear whether the homologous proteins in the HSSP database truly reflect the evolution of the corresponding fold family. It is also not clear whether the evolution of a particular family reaches equilibrium. Moreover, conservation for function and kinetics in addition to stability is also known to affect the sequence conservation. Indeed, we found that our model is able to recapitulate the native sequence entropies after excluding positions known to be responsible for apparent conserved functions and kinetics. The statistically significant correlations validate our Medusa/DMD method as a direct computational method to study protein evolution.

In the sequence alignment for detecting functional and structural homologs, there is a “twilight-zone” of sequence similarity [38,39], below which homologs are not well-defined. Empirically, this zone is in the range of 20%–30% sequence similarity. Interestingly, our Medusa/DMD simulations reproduces this range: above it the sequence similarity of core residues are higher than the rest of the protein, while below it the sequence similarity of core residues is often lower than the rest of the protein. Therefore, the sequence of core residues determines the protein homology. We also found in simulations that analogous proteins start to emerge once the structure deviates only 1–2 Å RMSD away from the reference protein. The emergence of the analogous sequences is not due to amino acid substitution kinetics in the sequence space. For each backbone structure (fixed-backbone simulation), we performed multiple independent simulations starting from random sequences. The resultant sequences for a fixed-backbone structure were always close homologs (identity >80%). Therefore, a fixed-backbone structure does not tolerate analogous sequences. The structural rearrangement (~1–2 Å RMSD) is necessary to accommodate the analogous sequences. These results corroborate the hypothesis that protein homologs and analogs can originate from the same ancestors [22], and that accumulative structural deviations, which are stabilized by accumulated mutations in the homologous families, lead to distinct packing in the protein core. The differentiation in the protein core composition of amino acids results in the formation of analogous protein families.

Efficient sampling of protein conformations is essential for a protein design algorithm with a flexible backbone. In the current study, we used DMD simulations to sample large-scale conformational changes and use a structure-relaxation method to sample small-scale conformations around a given structure. The sampling of conformational space by DMD currently separates from the Medusa redesign procedure. A more efficient approach is to directly integrate the fast dynamic sampling of conformations and the sequence redesign, which will require an all-atom DMD model (FD and NVD, unpublished data) with a Medusa-like DMD force field.

To rapidly find the optimal sidechain packing for a given protein backbone, a rotamer library-based search algorithm is often employed for the optimization process. Each discrete rotamer in a rotamer library is represented by the average dihedral angles and their standard deviations, derived from high-resolution PDB structures [44]. To model the dispersion of each rotamer state and also the possible strain between the sidechain and fixed backbone, additional divisions in the sub-rotameric space have been proposed [32]. However, such expansion of the rotamer library will require a large memory allocation, which in turn limits the study of large proteins. In our approach, we use a stochastic sidechain optimization in the sub-rotameric space, which does not require large memory allocation and has similar performance in recapitulating native sequences. We expect that our stochastic sidechain redesign algorithm will be able to simulate large proteins in the future applications. Therefore, our Medusa/DMD methodology can be applied to explore the sequence-structure space for proteins and protein complexes and can be applied to design proteins and protein-protein interactions.

## Materials and Methods

### Energy function and parameterization.

We modeled proteins using the united atom model, which includes all heavy atoms and polar hydrogen atoms. Similar to the RosettaDesign force field [28], the energy of a protein is computed as a linear sum of the follow terms:


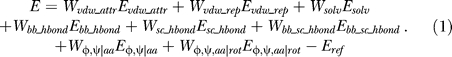


Here, *E_vdw_attr_*, *E_vdw_rep_* are the attractive and repulsive part of the VDW interaction, respectively; *E_solv_* is the solvation energy; *E_bb_hbond_*, *E_sc_hbond_* and *E_bb_sc_hbond_* are the hydrogen bond energies among backbones, among sidechains, and between backbones and sidechains, respectively. *E_φ,ψ|aa_* and *E_φ,ψ,aa|rot_* correspond to the internal energy for an amino acid (*aa*) in its rotamer state (*rot*) given the backbone dihedrals, phi (*φ*) and psi (*ψ*). *E_ref_* is the reference energy for the unfolded state. We use the weights (*W*) to estimate the contribution of each energy term to the total energy. The detailed description of each energy terms, the parameterization of weights (*W*), and energy calculation can be found in the Protocol S1.

### Fixed-backbone redesign using Medusa.

Given a protein's backbone, we used a Monte Carlo-based simulated annealing to search for low-energy sequences. Starting with a random sequence, we slowly decreased the system temperature. Using the Metropolis criterion, we accepted or rejected a trial mutation—either an amino acid substitution or a sidechain rotation—by computing the energy difference between the original and mutated sequences. During the last step of annealing, we performed a quenching simulation, in which a conjugate-gradient minimization was used to find the lowest energy in the sub-rotameric conformation of each trial rotamer. We accepted the substitution only if the minimum energy was lower than the current value. Due to the stochastic nature of the redesign algorithm, we performed multiple simulations. The resultant sequences for a given backbone are always close sequence homologs (sequence identity > 80%). We chose the lowest energy sequence as the optimal one.

For a given discrete rotamer state, there are associated dihedral angle variations with their standard deviations tabulated in the rotamer library [44]. Thus, a small deviation from the average dihedrals results in a different energy. To model the sidechain flexibility, we used the two following methods to sample the sub-rotameric space:

### Stochastic sidechain redesign.

In a stochastic sidechain redesign simulation, a trial rotamer is generated by assigning a random value to each sidechain dihedral angle, according to a Gaussian distribution within one standard deviation around its average value. The average dihedral angles of a rotamer and the corresponding standard deviations are taken from the rotamer library [32].

### Flexible sidechain redesign.

In the flexible sidechain redesign protocol, a minimization of the trial rotamer in its sub-rotamer space is performed for each trial substitution/mutation instead of in the last quenching step. The trial rotamer is accepted or rejected based on the energy difference between the minimum energy of the trial rotamer after minimization and the original energy.

### Generation of backbone conformation ensembles using DMD.

We performed DMD simulations to generate a large ensemble of backbone conformations for a given protein fold. Starting from a representative reference structure, we removed the sidechain atoms, except the beta-carbon (*C_β_*) atoms because our sequence search procedure starts from the backbone only and sidechains are subjected to substitution. To preserve the fold structure, we assigned a Gō potential [36,53] between *C_β_* atoms. In the Gō interaction model, the native contacts are favored over the non-native contacts by assigning attractive interaction potentials to the former. Thus, the conformations with low potential energies have their native contacts formed and are homologous to the reference structures. We constructed a homologous backbone conformation ensemble by collecting snapshots along the simulation trajectories from DMD simulations below folding transition temperatures, where the model protein stays folded with near-native potential energies.

### Protein redesign with backbone relaxation.

We iteratively performed fixed-backbone sequence redesign and structural relaxation to engineer an optimum sequence and structure for the starting conformation. During each iteration, we started from a randomly generated amino acid sequence and performed simulated annealing to identify the optimal sequence for the given initial backbone. During the annealing process, if the acceptance rate of a rotamer substitution at a given temperature was below a pre-defined value *P_relax_=* 0.05, we performed a conjugate-gradient based minimization to relax the backbone conformation with respect to the backbone dihedral angles using the same force field (Protocol S1). The cutoff acceptance rate, *P_relax_*, was chosen such that amino acid sidechains at this temperature have no severe VDW clashes, and the packing of the core is compact. At the end of the iteration, we randomly reassigned amino acid sequence for the next iteration. After a maximum number iterations, the sequence and relaxed structure with the lowest energy was accepted.

In the structure-relaxation step, a conjugate-gradient based minimization algorithm was used to minimize the total energy with respect to the backbone dihedrals Φ, Ψ and Ω. The dihedral angle Ω models the strain energy of the peptide plane and assumes a Gaussian distribution with an average value of 179^°^ and standard deviation of 5.6^°^ [32]. The energy gradients for the backbone dihedral angles were computed using an efficient recursive calculation methods as in Ref. [54].

### Protein core.

Following previously published methods [32], we defined protein cores as the residues with >20 residue-wise contacts. A contact between a residue pair is defined once the distance between corresponding beta carbon atoms (*C_α_* for GLY) is within 10 Å.

### Sequence entropy.

To quantify the sequence conservative profile for a given residue position, we defined the sequence entropy *S*(*i*) as: 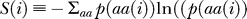 . Here, (*aa*(*i*)) is the probability of finding an amino aicd *aa* at the *i*th position in a given homologous sequence ensemble after weighting all sequences by the Henikoff position-based weighting algorithm [55], which we used to reduce the redundancy in sequences and the apparent “conservation” due to the number of available sidechain rotamers.

## Supporting Information

Figure S1 The Protein Sequence/Structure Optimization with Backbone Relaxation The sequence identities, backbone deviation, and the energy at the end of each iteration are shown.
(94 KB DOC)

Protocol S1 Supporting Materials and Methods (73 KB DOC)

Table S1 The Weight of Each Energy Term (Materials and Methods) (44 KB DOC)

Table S2 The Average Native Sequence Recapitulation Rate between Native and Redesigned Sequences, with Different Subsets of Energy Terms (28 KB DOC)

## Abbreviations

DMD: discrete molecular dynamics
HPR: histidine-containing phosphocarrier protein
HSSP: homology-derived secondary structure of proteins
PDB: protein databank
RMSD: root-mean-square-deviation
VDW: van der Waals
