# Correction: Emergence of Protein Fold Families through Rational Design

**DOI:** 10.1371/journal.pcbi.0020149

**Published:** 2006-10-27

**Authors:** Feng Ding, Nikolay V Dokholyan

In *PLoS Computational Biology,* volume 2, issue 7: DOI: 10.1371/journal.pcbi.0020085

The references to the figure parts in the legend of figure 3 were incorrect. The correct caption is as follows:

**Figure 3.** The Sequence Identity for the Constructed Homologous Structures

Three different protein folds are studied: HPR domain (A,D), ROSSMAN fold (B,E), and SH3 domain (C,F). (A,B,C) The sequence identities of the redesigned proteins using the flexible-backbone design simulation are presented as the function of the backbone-RMSD from the reference protein. (D,E,F) The sequence identity of the core is also plotted against the overall sequence identity. The “twilight zone” of sequence identity (20%–30%) corresponds to regions between horizontal (A,B,C) or vertical (D,E,F) lines.

