# Supplementary material for: Emergence of Protein Fold Families through Rational Design
Source: PLoS Comput Biol. 2006 Jul 7;2(7):e85. doi: 10.1371/journal.pcbi.0020085 (PMC1487181; doi:10.1371/journal.pcbi.0020085)
Supplement: Supplementary file 1 [file pcbi.0020085.sd001.doc]

**Supplementary materials**

**Energy terms.** The energy of a given sequence and structure is evaluated by the following terms:

*Van der Waals Potentials (E_vdw_attr_, E_vdw_rep_)*. We use a standard 12-6 Lennard-Jones (LJ) potential to model the attractive portion of Van der Waals interactions. For simplicity, we use a linear extrapolation to model VDW repulsion to damp the quick increase of regular LJ repulsions.

$\begin{aligned} E_{\text{vdw}-\text{attr}}=\sum_{i,j>i} 4\varepsilon_{\text{ij}}[({\sigma_{\text{ij}}}/{r_{\text{ij}}})^{\text{12}}-({\sigma_{\text{ij}}}/{r_{\text{ij}}})^{6}],r_{\text{ij}}>\sigma_{\text{ij}} \\ E_{\text{vdw}_{\text{rep}}}=\left\{ \begin{matrix} \sum_{i,j>i} 4\varepsilon_{\text{ij}}[({\sigma_{\text{ij}}}/{r_{\text{ij}}})^{\text{12}}-({\sigma_{\text{ij}}}/{r_{\text{ij}}})^{6}],\alpha_{\text{cutoff}}\sigma_{\text{ij}}<r_{\text{ij}}\leq\sigma_{\text{ij}} \\ \begin{aligned} K_{\text{slope}}r_{\text{ij}}+4\varepsilon_{\text{ij}}(\alpha_{\text{cutoff}^{-\text{12}}}-\alpha_{\text{cutoff}^{-6}})-\alpha_{\text{cutoff}}K_{\text{slope}}\sigma_{\text{ij}},r\leq\alpha_{\text{cutoff}}\sigma_{\text{ij}} \\ \text{Here},\alpha_{\text{cutoff}}=0\text{.}\text{93};K_{\text{slope}}=-\text{24}\varepsilon_{\text{ij}}(2\alpha_{\text{cutoff}^{-\text{13}}}-\alpha_{\text{cutoff}^{-7}})/{\sigma_{\text{ij}}} \end{aligned} \end{matrix} \right. \\ \varepsilon_{\text{ij}}=\sqrt{\varepsilon_{i}\varepsilon_{j}};\sigma_{\text{ij}}=\sigma_{i}+\sigma_{j} \end{aligned}$.	(S2)

Here, *r_ij_* is the distance between two atoms *i* and *j*. The energy parameters *ε*, *σ* are taken from the CHARMM19 force field of united atoms [1].

*Solvation energy (E_solv_)*. We approximate the solvation energy with EEF1 model proposed by Lizaridis and Karplus [2]**:**

$\begin{aligned} E_{\text{solv}}=\sum_{i,j>i} [-\frac{2{\Delta G}_{i}^{\text{free}}}{4\pi\sqrt{\pi}\lambda_{i}r_{\text{ij}}^{2}}\text{exp}(-x_{\text{ij}}^{2})V_{i}-\frac{2{\Delta G}_{j}^{\text{free}}}{4\pi\sqrt{\pi}\lambda_{j}r_{\text{ij}}^{2}}\text{exp}(-x_{\text{ji}}^{2})V_{j}] \\ x_{\text{ij}}=(r_{\text{ij}}-1\text{.}\text{12}\sigma_{i})/{\lambda_{i}};x_{\text{ji}}=(r_{\text{ij}}-1\text{.}\text{12}\sigma_{j})/{\lambda_{j}} \end{aligned}$.	(S3)

Here, the parameters of reference solvation energy (Δ*G^free^*), volume of atoms (V), correlation length (λ), and atom radius (σ) are taken from Lizaridis and Karplus. We do not include the intrinsic solvation energy [2] of each atom, since the sum of these terms for each amino acid is a constant that can be incorporated into the reference energies. The calculation of van der Waals and solvation energies uses a distance cutoff of 9Å.

To avoid the over-estimation of the solvation energy when the atom pair *i,j* are too close, we define a cutoff distance *d_ij_=0.95σ_ij_*. When the distance *r_ij_* is smaller than *d_ij_*, the solvation energy will be a constant of *E*(*d_ij_*).

*Hydrogen bond interactions (E_bb_hbond_, E_sc_hbond_ and E_bb_sc_hbond_).* To model the electrostatic interactions by hydrogen bond formation, we use an orientation-dependant hydrogen bond model proposed by Kortemme and Baker [3]. The energy of backbone-backbone (*E_bb_hbond_*), sidechain-backbone (*E_bb_sc_hbond_*) and sidechain-sidechain (*E_sc_hbond_*) hydrogen bonds were derived from the statistics of naturally-occurring hydrogen bonds in the PDB database.

In the hydrogen bond model, the optimal distance between donor and acceptor in a hydrogen bond is approximately 2.95Å, which is often much smaller than the VDW radii between these atoms in CHARMM19 force field. To favor the formation the hydrogen bonds, we assign the VDW radii between all the potential donor and acceptor pairs *σ_donor-acceptor_ = 2.95/2^1/6^*Å.

### *Backbone dependent internal energies (E_φ.ψ|aa_, E_φ.ψ,aa|rot_).* We use a backbone dependent rotamer library from Dundrack and Cohen [4] to perform the search of optimal sidechian packing. To account for the internal energy for the rotamer state, we use the log normal of the natural occurring probability for a specific rotamer in the PDB, derived by Dunbrack et al., as its internal energy:

$E_{\varphi,\psi,\text{aa}|\text{rot}}=-\text{ln}(P_{\varphi,\psi,\text{aa}|\text{rot}})$.	(S4)

To account for the variation of amino acids in a specific backbone *φ.ψ* conformation, we also include the internal energy for different amino acids. We compute from the non-redundant PDB library the natural occurring probability of different amino acids in a given backbone dihedral angles *φ,ψ*, *P_φ,ψ|aa_*. To be consistent with the Dunbrack backbone dependent rotamer library, we use a 10 degree bin size for *φ,ψ* angles. We use the log normal of *P _φ,ψ|aa_* as the energy contribution:

$E_{\text{phi},\text{psi}|\text{aa}}=-\text{ln}(P_{\text{phi},\text{psi}|\text{aa}})$.	(S5)

Thus, *E_φ,ψ|aa_* and *E_φ,ψ,aa|rot­_* will account for the internal energy between the (*aa,rot*) pair in the sidechain with the neighboring backbone atoms. In order to avoid overcounting in the energy calculation, the calculation of van der Waals and solvation energies will exclude interactions between *i*th residue and its neighboring backbone atoms *C_αi-1_*, *C_i-1_*, *O_i-1_*, *N_i+1_*, *HN_i+1_*, and *C_αi+1_*.

*Reference energy (E_ref_).* We use an empirical energy for each amino acid to model the unfolded the unfolded state.

$E_{\text{ref}}=\sum_{i} W(\text{aa}_{i})=\sum_{\text{aa}} n_{\text{aa}}W(\text{aa})$.	(S6)

**Weights determination**. Since the energy terms we use are taken from different sources, we use weighted coefficients, *W*, to scale the contribution for each of them to total energy. In order to determine these coefficients, we use a procedure similar to the one proposed by Kuhlman and Baker [5]. For a given position, we substitute the native amino acid/rotamer, (*aa_nat_,rot_nat_*), with all possible amino acid/rotamer (*aa,rot*). For each (*aa,rot*), we compute the corresponding energy, *E*(aa,rot). We use a training set of 34 high-resolution x-ray crystal structures and maximize the probability to observe the native (*aa,rot*) at each position,

$P(aa_{nat},rot_{nat})=\exp(-E(aa_{nat},rot_{nat}))/\sum_{(aa,rot)} \exp(-E(aa,rot))$.	(S7)

Then, we maximize the product of $\prod_{\text{prot},i} P(\text{aa}_{\text{nat}},\text{rot}_{\text{nat}})_{\text{prot}}^{i}$at each position for all proteins in the training set. In addition, we introduce a term into the optimization function to minimize the deviation of the twenty amino acids distributions between the prediction and observation. For each position, we select the amino acid with lowest energy as the prediction.

$S=-\sum_{\text{prot},i} \text{ln}(P(\text{aa}_{\text{nat}},\text{rot}_{\text{nat}})_{\text{prot}}^{i}+\sum_{\text{aa}} [n_{\text{aa}}^{\text{pred}}-(\sum_{\text{aa}'} n_{\text{aa}'}^{\text{pred}})p_{\text{aa}}^{\text{PDB}}]^{2}$.	(S8)

Here, $n_{\text{aa}}^{\text{pred}}$ is the number of amino acid *aa* that are predicted for all the positions in the training set. $P_{\text{aa}}^{\text{PDB}}$represents the distribution of twenty amino acids in PDB.

*Sub-rotamers.* For a given discrete rotamer state, there is an allowed dihedral angle variations with its standard deviations tabulated in the rotamer library [4]. Thus, a small deviation from the average dihedrals results in a different energy. To compute the energy contributions for a specific rotamer, we use the lowest energy sidechain conformation with sidechain dihedral angles limited within one standard deviation around the average value. We confine dihedral angles by assigning a half-harmonic potential near the two edges:

$E_{\text{confinement}}(\theta)=\left\{ \begin{matrix} E_{0}(\theta-(\overline{\theta}-\Delta\theta))^{2},\theta<\overline{\theta}-\Delta\theta\\ 0,\overline{\theta}-\Delta\theta<\theta<\overline{\theta}+\Delta\theta\\ E_{0}(\theta-(\overline{\theta}+\Delta\theta))^{2},\theta>\overline{\theta}+\Delta\theta\end{matrix} \right.$.	(S9)

Here, *E_0_* is a constant chosen be 100 kcal/mol to impose a strong penalization for deviations. Parameters $\overline{\theta},\Delta\theta$are the average sidechain dihedral angle and its standard deviation taken from Dundrack rotamer library. We use a conjugate-gradient based minimization algorithm to find the lowest energy sub-rotamer conformations and then to compute the corresponding energy terms.

To perform the minimization, we start with a pre-assigned set of weights to calculate the energies for each rotamer. Based on computed energies, we determine the optimal weights by minimizing the scoring function of Eq. (S8). The new weights are used for the next round of calculations. With such an iterative procedure in determining the optimal weights, we find that the weights rapidly converge after two iterations. We list the determined weights in Table S1.

**Energy calculation.** In our protein model, each residue position is fitted with all twenty amino acid types, although only one amino acid type is active when the energy is evaluated. A point mutation is achieved by simply deactivating and activating the related amino acids at a given position. The rotamer state of an amino acid is obtained by appropriate rotations of the sidechain dihedral angles to the values given in the rotamer library.

To facilitate the calculation of energy, we put the protein into a three dimensional grid, where each cell is a cubic box with the dimension equal to the interaction cutoff distance, beyond which the interaction is zero. Each cell keeps a list of atoms within it. Thus, the energy evaluation for a given atom is only between itself and atoms in the neighboring 3^3^=27 cells. The hydrogen bond interaction has a maximum interaction distance of 3 Å [3], shorter than that of the VDW and solvation interaction, which is set as 9 Å in our study. Therefore, we use two different grids for hydrogen bond interaction and for VDW + solvation interactions, respectively: for VDW and solvation interactions we use a grid with a cell-dimension of 9 Å; for hydrogen bond interaction, we use a grid with a cell-dimension of 3 Å.

Reference List

1.	Neria E, Fischer S, Karplus M (1996) Simulation of activation free energies in molecular systems. Journal of Chemical Physics 105: 1902-1921.

2.	Lazaridis T, Karplus M (1999) Effective energy function for proteins in solution. Proteins 35: 133-152.

3.	Kortemme T, Morozov AV, Baker D (2003) An orientation-dependent hydrogen bonding potential improves prediction of specificity and structure for proteins and protein-protein complexes. J Mol Biol 326: 1239-1259.

4.	Dunbrack RL, Jr., Cohen FE (1997) Bayesian statistical analysis of protein side-chain rotamer preferences. Protein Sci 6: 1661-1681.

5.	Kuhlman B, Baker D (2000) Native protein sequences are close to optimal for their structures. Proc Natl Acad Sci U S A 97: 10383-10388.
