# Supplementary material for: Emergence of Protein Fold Families through Rational Design
Source: PLoS Comput Biol. 2006 Jul 7;2(7):e85. doi: 10.1371/journal.pcbi.0020085 (PMC1487181; doi:10.1371/journal.pcbi.0020085)
Supplement: Supplementary file 2 [file pcbi.0020085.sg001.doc]

**Figure S1**. The protein sequence/structure optimization with backbone relaxation. The sequence identities, backbone deviation, and the energy at the end of each iteration are shown.
