# Supplementary material for: Emergence of Protein Fold Families through Rational Design
Source: PLoS Comput Biol. 2006 Jul 7;2(7):e85. doi: 10.1371/journal.pcbi.0020085 (PMC1487181; doi:10.1371/journal.pcbi.0020085)
Supplement: Supplementary file 3 [file pcbi.0020085.st001.doc]

**Table S1**. The weight of each energy terms (Methods).

| Energy wrights | The parameterized value |
| --- | --- |
| *W_VDW-attr_* | 1.00 |
| *W_VDW-rep_* | 0.87 |
| *W_solv_* | 0.73 |
| *W_bb-hbond_* | 2.00 |
| *W_bb-sc-hbond_* | 1.63 |
| *W_sc-hbond_* | 1.84 |
| *W_φ,ψ\|aa_* | 0.63 |
| *W_φ,ψ,aa\|rot_* | 0.62 |
| *W_CYS_* | 2.00 |
| *W_MET_* | -0.49 |
| *W_PHE_* | 2.74 |
| *W_ILE_* | 0.52 |
| *W_LEU_* | 0.57 |
| *W_VAL_* | -0.07 |
| *W_TRP_* | 4.32 |
| *W_TYR_* | 2.46 |
| *W_ALA_* | -1.07 |
| *W_GLY_* | -1.41 |
| *W_THR_* | -0.86 |
| *W_SER_* | -1.20 |
| *W_GLN_* | -0.22 |
| *W_ASN_* | -1.15 |
| *W_GLU_* | -2.16 |
| *W_ASP_* | -2.43 |
| *W_HIS_* | 0.88 |
| *W_ARG_* | -0.04 |
| *W_LYS_* | -1.33 |
| *W_PRO_* | 0.94 |
