# Supplementary material for: Emergence of Protein Fold Families through Rational Design
Source: PLoS Comput Biol. 2006 Jul 7;2(7):e85. doi: 10.1371/journal.pcbi.0020085 (PMC1487181; doi:10.1371/journal.pcbi.0020085)
Supplement: Supplementary file 4 [file pcbi.0020085.st002.doc]

**Table** **S2**. The average native sequence recapitulation rate between native and redesigned sequences with different subsets of energy terms. To turn off an energy term, we set the corresponding weight, W, zero. The detail description of different weights can be found in Method section.

| **Control Simulations** | **Core** | **Overall** |
| --- | --- | --- |
| Full coefficient set {*W*} | 58.6% | 37.8% |
| *W_solv_=0* | 15.2% | 8.1% |
| *W­_solv_=0; W­_ref_=0* | 13.0% | 12.3% |
| *W_solv_=0; W_HB_=0* | 16.0% | 11.9% |
| *W­_solv_=0; W_HB_=0; W­_ref_=0* | 13.3% | 20.1% |
| *W_φ.ψ\|aa_=0; W_φ.ψ,aa\|rot_=0* | 11.6% | 19.8% |
